# Development of the capacity to suffer in embryos and chicks: a systematic review of relevant studies

**DOI:** 10.3389/fvets.2025.1698528

**Published:** 2025-10-23

**Authors:** Jenny L. Mace, Andrew Knight

**Affiliations:** ^1^Centre for Ethics, Philosophy and Public Affairs, University of St Andrews, St Andrews, Fife, United Kingdom; ^2^Mace Animal Welfare, Dunfermline, Fife, United Kingdom; ^3^School of Veterinary Medicine, College of Environmental and Life Sciences, Murdoch University, Murdoch, WA, Australia; ^4^School of Environment and Science, Griffith University, Nathan, QLD, Australia; ^5^Animal Welfare Research Group, Faculty of Health and Wellbeing, University of Winchester, Winchester, United Kingdom

**Keywords:** chicks, chick embryos, pain, capacity to suffer, hatcheries, chick welfare, chicken welfare

## Abstract

Approximately 1.8 billion chicks are hatched worldwide in commercial hatcheries every month. A typical commercial hatchery is a high-speed and stressful environment. Not only is chick welfare impacted while at the hatchery, but also chickens’ early life experiences can have long-lasting impacts on their welfare once they leave the hatcheries. Additionally, chick embryos may have the capacity to experience stress and pain. This study systematically reviewed recent scientific studies exploring the starting point for the capacity to suffer in chicks and chick embryos. It found that the capacity to suffer (i.e., to experience pain, distress, or other prolonged negative welfare states) may commence by embryonic day 18—three days before hatching—and likely earlier. Based on this, serious and widespread welfare problems may exist for the 1.8 billion chicks hatched in hatcheries globally every month.

## 1 Introduction

**Figure 1:**
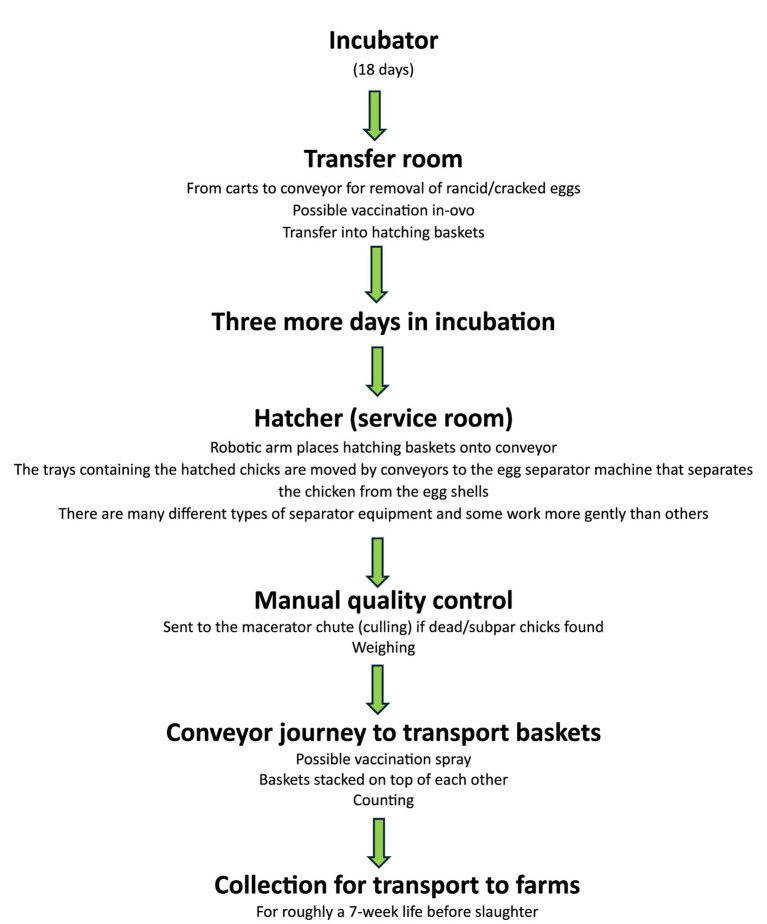
Representative stages in a commercial “broiler” chick hatchery, adapted from content in the USDA’s Poultry Industry Manual (4) (p. 16–18) and observations by Knowles et al. (3).

Globally, an estimated 1.8 billion chicks are hatched every month primarily to serve the chicken meat and egg industries—but also to serve backyard chicken keepers, scientists, and other more fringe users of chickens (1). This amounts to roughly 900 million chicks per month in the USA alone (2) (p. 15). A typical hatchery is highly automated and processes chicks through the stages of hatching, conveying, sexing (for layers), maceration (especially for males from laying breeds which are unwanted), vaccination, and beak trimming (for layers)—up to roughly seven stages, as depicted in Figure 1 (3, 4). A hatchery conveyor belt can have an acceleration of up to 920 m/s^2^, and drops of up to 55 cm (5) (pp. 275–276), with ambient noise levels of up to 70 dB and both mechanical and manual handling (6) (p. 136, 138). The largest and most modern hatcheries can process up to 100,000 chicks per hour, amounting to 4 million per week [e.g., see (7)]. Processing between 1 and 2 million chicks per week is not uncommon (3, 8).

The culling of male chicks in hatcheries is gaining increasing attention and even being outlawed in some European countries such as Germany (9) (p. 30). However, the welfare concerns about chicks and potentially chick embryos in hatcheries extend beyond the culling of male chicks, as raised by Knowles et al. (3), RSPCA (10), and Animal Equality USA (46) in recent undercover footage. The concerns span avoidable injuries and deaths from the use of unsuitable equipment, ill-maintained machinery, and insufficient staff training or oversight. To assess the potential for suffering of chicks and chick embryos processed at typical hatcheries, this study systematically sourced and analyzed recent scientific evidence regarding the stage of life at which chicks begin to feel pain and distress (i.e., the capacity to suffer). This will also be of great importance for discussions surrounding the latest embryonic stage at which in-ovo sex identification should take place to enable the painless killing of male embryos.

For this study, widely accepted definitions of pain, suffering, distress, and welfare in nonhuman animals were used, as follows: “Pain is an unpleasant sensory and emotional experience associated with, or resembling that associated with, actual or potential tissue damage” [International Association for the Study of Pain (IASP) (11); p. 2], and “Suffering is one or more bad feelings continuing for more than a short period” (12) (p. 60). To define *di*stress, stress must first be defined: “Stress is the biological response elicited when an individual perceives a threat to its homeostasis” (13) (p. 1). Moberg and Mench (13) then define *di*stress as occurring “when the stress response threatens an individual’s wellbeing” (p. 1). The welfare of an animal is a state which describes how well the animal is coping with his/her environment (12) (p. xiv). While pain and suffering are conceptually defined in the literature, their operationalization in studies involving chick embryos requires careful consideration. Given the absence of verbal communication and overt behavioral expression, researchers rely on indirect indicators such as the maturation of neural pathways (e.g., thalamocortical connections), electrophysiological responses to noxious stimuli, and the presence of coordinated motor reactions (14–16). These proxies are interpreted within a developmental framework, acknowledging that the capacity to suffer likely depends not only on nociceptive processing but also on integrative brain functions associated with affective experience (15). Therefore, in this review, we consider suffering as a multidimensional construct that is inferred from converging neurophysiological and behavioral evidence across embryonic stages.

## 2 Methodology

We conducted a systematic review of relevant scientific studies exploring the time point at which chicks and chick embryos start to have the capacity to suffer. We chose a systematic review in preference to other forms of evidence. The personal and potentially subjective opinions of experts are considered less reliable than more objective scientific literature analyses (17). Narrative literature reviews often focus on a subset of the literature, based on availability or author choice. These can create conscious or unconscious biases during the selection and inclusion of scientific evidence (18). In contrast, systematic literature reviews aim to minimize bias by identifying and analyzing all relevant studies on a specific topic, using robust and transparent criteria. These are considered to provide evidence of the greatest level of reliability when exploring scientific topics, and their use for such purposes is considered best practice (17–19). Systematic reviews require a transparent detailed search strategy and defined inclusion and exclusion criteria before starting the review. The identification process often utilizes bibliographic scientific literature databases, but can also be supplemented by checking reference lists or manually searching key journals to increase reliability and completeness.

The Preferred Reporting Items for Systematic Reviews and Meta-Analyses (PRISMA) guidelines provide best practice guidelines for conducting systematic reviews (19). Accordingly, the PRISMA guidelines (2020 updated version) were adhered to in the present study. The following leading bibliographic scientific literature databases were used: Web of Science All Databases and Scopus. This concurs with current recommendations regarding the selection of databases for systematic reviews, namely, the use of at least three verified databases (20). Because the All Databases version of Web of Science comprises its Core Collection in addition to Medline and numerous supplementary databases, this fulfills and surpasses these criteria. Additionally, Web of Science Core Collection, Medline, and Scopus have recently been designated as “principal” databases that should be used for systematic reviews (21). They are also all either multidisciplinary or biomedically oriented, which is suitable for the field of enquiry at hand.

The following search string for all databases was devised after extensive piloting of different search strings, and following an initial review of key literature to guide keyword choices: (chick* OR galliform* OR “gallus gallus” OR “gallus domesticus” OR fowl OR bird OR avian OR poultry) AND (in-ovo OR embryo OR fetus OR foetus OR hatchling OR young OR neonatal OR newly-hatched OR day-old) AND (pain OR nocicep* OR suffer* OR distress OR discomfort) AND (stage OR neuron*) AND (development* OR incubation). This provided an appropriate balance of both sensitivity (ensuring key results were not missed) and specificity (ensuring irrelevant results were not included), as described by Bramer et al. (22). Our pilot review found that all “AND” components of our search terms were present within the abstracts of key studies we knew we needed to retrieve; thus, to ensure sufficient specificity, we required all of these components to be present by using “AND.” One digital skills librarian at the University of Winchester, UK, also confirmed the technical suitability of the search string, and recommended checking for other synonyms via EBSCOhost (a very large online research platform providing access to bibliographic databases and search features) and a thesaurus, which was done.

**Figure 2:**
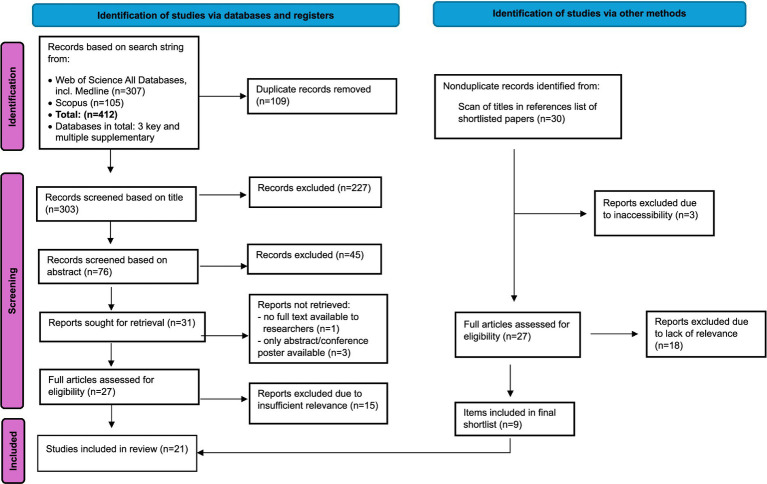
PRISMA flow diagram, adapted from Page et al. (19), used under CC BY 4.0 license.

Both databases were searched on 24th September, 2024. A flow diagram summarizing the systematic review stages is provided in Figure 2. No further refinements or exclusions were applied in the searches. Common reasons for excluding items were that studies *only* focused on adult chickens, on a different species, or on genetics or another insufficiently relevant topic. If it was unclear whether a title was relevant or not, it was retained for a review of the abstract or full paper at subsequent analysis stages; for instance, papers about sexing of chick embryos were retained in case they included information about neurological development relevant to pain perception. The reference lists of the final shortlist of records were also reviewed in case any additional items of importance were present. There were two shortlisted papers written in German, but we collectively possessed advanced German skills, so these were retained.

The shortlisted items were then summarized into Table 1. Essential characteristics and findings were then tallied and analyzed. Consistent with systematic review best practice, the reliability and relevance of the shortlisted items were then assessed and indicated using red/amber/green colors to convey low/medium/high levels, respectively. However, a reliability analysis for each shortlisted study is not an *essential* component of the PRISMA (19) checklist. Thus, it should be noted, that due to resource limitations, the reliability analyses were based on the subjective assessment of the first author, primarily considering methodological factors (e.g., sample size, starting day of embryonic analysis, and any missing details), but also clarity of the write-up. Green was awarded if no problems or weaknesses were detected, amber if one to three weaknesses were detected, and red if more than three weaknesses were detected (or two major ones). The relevance of the items was likewise based on assessment by the first author. This centered on the relevance of the research questions of each shortlisted item. The scientific information collected was then analyzed to answer the research question: *At what stage of development does the chick embryo begin to have the capacity to suffer?*

**Table 1:** Summary of systematic review results focusing on the question: at what stage of development do chick/chick embryos have the capacity to suffer?

No.	Author(s); date	Year	Title	Study type	Aim/research question	Key content/result(s)	Reliability	Relevance
1	Weiss et al.	2023	Nociception in chicken embryos, part I: Analysis of cardiovascular responses to a mechanical noxious stimulus	Physiology	Determine when the capacity of nociception emerges during embryonic development in chickens.	“Infiltration anesthesia with the local anesthetic lidocaine significantly reduced the response of MAP [mean arterial pressure] on **ED18**, so the measured cardiovascular changes may be interpreted as nociceptive responses” (p. 1). “[I]t must be assumed that a nociceptive cardiovascular response is possible in individual embryos at **ED15**″ (p. 11).		
2	Kollmansperger et al.	2023	Nociception in chicken embryos, part II: Embryonal development of electroencephalic neuronal activity in-ovo as a prerequisite for nociception	Neuroscience	Evaluate the ontogenetic point at which noxious stimuli could potentially be perceived/processed in the brain *in ovo*.	“The onset of physiological neuronal signals could be determined at developmental **day 13** … The results suggest developmental **day 13** as the earliest embryonal stage being able to receive and process nociceptive stimuli” (p. 1).		
3	Süß et al.	2023	Nociception in chicken embryos, part III: Analysis of movements before and after application of a noxious stimulus	Behavior	Examine the movements of developing chicken embryos with the aim of identifying behavioral responses to a noxious stimulus.	“After the application of the mechanical stimulus, a significant increase in beak movement was identified in 15- to 18-day-old embryos. In younger embryos, no behavioral changes related to the noxious stimulus were observed. The presented results indicate that noxious mechanical stimuli at the beak base evoke a nocifensive reaction in chicken embryos starting at embryonic **day 15**” (p. 1).		
4	Douglas et al.	2018	Pain in Birds: The anatomical and physiological basis	Review	To give an updated account of the anatomical and physiological basis of pain in birds	“…that pain increases metabolism in an area of the avian brain possibly rich in opioid receptors” (p. 22). “In the chicken forebrain and midbrain, μ receptors were most prevalent and are detectable in chick embryos at **10** days of age” (p. 23). Time point for onset of suffering cannot be inferred.		
5	Deutscher Bundestag	2017	On the sensation of pain in chicken embryos [Zum Schmerzempfinden von Hühnerembryonen]	Review	To review recent literature regarding the point at which chick embryos can feel pain.	There is a consensus of no pain perception earlier than **E7**, and of pain perception at the latest from **E15**. But there is no consensus regarding if pain perception may commence between these times [translated] (p. 6).		
6	Nicol	2015	Development of the Brain and Behavior	Review	To review large body of research examining brain /behavior development in chickens (emerging from chick models, used for broader vertebrate understanding).	“Peak neuron formation at **E8**; peak in synaptic connections between neurons in forebrain at **E15**” (p. 37).		
7	Liu et al.	2014	Semaphorin 5B is a repellent cue for sensory afferents projecting into the developing spinal cord	Cytology	To investigate inhibitory cues that create the “waiting period” preceding sensory axon projection onto the gray matter of the chick embryo spinal cord.	“It has been well described that sensory collaterals do not enter the gray matter until **E6** (st29) and then project to specific laminae targets by **E9** (st35) according to their sensory modality” (p. 1941). “We found that Sema5B is present in the chick spinal cord as early as **E3**, the developmental period when the first sensory axons are targeting the DREZ [dorsal root entry zone]” (p. 1944). Time point for onset of suffering cannot be inferred.		
8	Bellairs et al.	2014	The Atlas of Chick Development	Review	To review the development of the chick	“By stage 16 [**<E3**], spinal nerves have developed and by stage 22 (**day 3.5–4**), regions of “gray” and “white” matter are recognizable … Dorsal and ventral horns can be seen in the gray matter from **day 7** and glial cells in the white matter” (p. 54). Time point for onset of suffering cannot be inferred.		
9	George et al.	2010	Patterned assembly and neurogenesis in the chick dorsal root ganglion	Cytology	Investigate the orchestration and emergence of the chick dorsal root ganglion.	“Small-diameter TrkA+ afferents that mediate pain and temperature sensation are primarily born during the second wave of neurogenesis that begins at St.25 [**E4.5**] and persists for the next 48 h” (p. 405). Time point for onset of suffering cannot be inferred.		
10	Mellor et al.	2007	Birth and hatching: Key events in the onset of awareness in the lamb and chick	Review	Does the developing domestic chick become conscious/aware before or after hatching?	“These observations are consistent with the chick not being capable of exhibiting any cerebral state that resembles awareness earlier than **Day 17**. Thereafter **until hatching**, although the chick’s brain might be capable of supporting states of awareness, especially as hatching approaches, the EEG evidence above suggests that the chick remains in sleep-like states of unconsciousness for most, and probably all, of that period. Moreover, during hatching itself additional EEG evidence suggests that sleep-like unconsciousness persists” (p. 54).		
11	Necker	2005	Embryonic development of choline acetyltransferase and nitric oxide synthase in the spinal cord of pigeons and chickens with special reference to the superficial dorsal horn	Histology	To study embryonic development in precocial species.	“The spinal gray substance of the **E14** chicken embryo already shows the adult-like organization with lamina II and lamina III lying side by side” (p. 150). “Neurons of lamina II are known to be involved in nociception and there is some evidence that NO contributes to the modulation of nociceptive information” (p. 151). “The late appearance of both ChAT [choline acetyltransferase] and NOS [nitric oxide synthase] in the superficial dorsal horn during ontogeny suggests that these modulatory systems are needed only when the animals start to live on their own, which is the case at the **time of hatching** in precocial birds” (p. 153).		
12	Bernardini et al. (47)	1998	Neuronal and non-neuronal cell populations of the avian dorsal root ganglia express muscarinic acetylcholine receptors	Cytology	Investigate the distribution of muscarinic acetylcholine receptors at E12, E18, and post hatching.	“This indicates that at this developmental stage [**E12**] although peripheral fibers have already reached their targets, mAChRs are not yet involved in the transduction of sensory stimuli” (p. 375). Time point for onset of suffering cannot be inferred.		
13	Koltzenburg et al.	1997	Receptive properties of embryonic chick sensory neurons innervating skin	Neuroscience	Describe new in-vitro skin nerve preparation from chick embryos.	“It is clear from our data, that by **E17**, and probably earlier, the mechanical thresholds of putative nociceptors are sufficiently low to be activated by embryonic limb movements, which start around **E7** … the receptive properties of nociceptors undergo gradual maturation in the post-hatching period. Thus the responses of C fibers to noxious heat become more dynamic and robust” (p. 2567). “[A]t the **time of hatching**, there is still considerable plasticity in the capacity of neurons to respond to noxious stimuli” (p. 2566) “[N]ociceptive afferents are likely to be capable of activating spinal cord NMDA receptors [involved in pain recognition] during the embryonic period from **E12** onwards, the time at which lamina II begins to be innervated in the chick” (p. 2567). “It is possible that these [nociceptive] receptors have even lower mechanical thresholds earlier in development because their receptive thresholds continue to rise after **E17**” (p. 2567).		
14	Rosenbruch	1997	The sensitivity of chicken embryos in incubated eggs [Zur Sensitivität des Embryos im bebrüteten Hühnerei]	Review	To review the literature on pain sensitivity in chicken embryos.	“The sensitivity develops stepwise, beginning around **day 7** of incubation” (p. 111). “The complete differentiation of the corresponding areas of the central nervous system are however only completed on the **18**^**th**^ **day**” (p. 112).		
15	Steeves et al.	1994	Permissive and restrictive periods for brainstem-spinal regeneration in the chick	Cytology	Cellular factors contributing to functional axonal regeneration	“By **E11** of normal embryonic development, the distribution and number of retrograde labeled brainstem-spinal neurons is equivalent to those labeled in a chick after hatching” (p. 245). Time point for onset of suffering cannot be inferred.		
16	Covell et al.	1989	Embryonic development of the chick primary trigeminal sensory-motor complex	Cytology	To outline all developmental stages of the chick embryo’s sensory-motor complex.	“Neuritic processes initially extend from the somata of ophthalmic placodal sensory neurons both toward the CNS and peripherally at approximately the same time, beginning at stage 15 [**<E3**]” (p. 501) “Neural crest cells do not initiate axon formation until at least **day 4** to 5” (p. 488). “Labeled terminal arborizations of descending trigeminal afferents are first visible at stage 22 and are evident along the entire descending and proximal ascending tracts by stage 27 [**E5**]” (p. 488). Time point for onset of suffering cannot be inferred.		
17	Davis et al.	1989	Development of central projections of lumbosacral sensory neurons in the chick	Cytology	Examine the development of central projections of sensory neurons in lumbosacral dorsal root ganglia	“… primary afferents reach the spinal cord by stage 23. Afferent axons extend in the primordium of the dorsal funiculus for several segments rostral and caudal to their segment of entry for over 24 h before invading the gray matter at stage 28 (**E6**). Sensory fibers grow into the vicinity of motoneuron dendrites by stage 32 (**E7.5**), about the time that reflexes and apparent monosynaptic EPSPs can first be elicited. Dense projections into the dorsal laminae of the spinal cord, presumably representing cutaneous afferents, appear somewhat later, at about stage 39 (**E13**), when the segmental projection pattern begins to resemble the mature pattern” (p. 556). Time point for onset of suffering cannot be inferred.		
18	New et al.	1986	Distribution and ontogeny of SP, CGRP, SOM, and VIP in chick sensory and sympathetic ganglia	Histology	“Study further the relationship between neuron position, modality, and peptide expression within chick peripheral ganglia”	“…substance P (SP) and calcitonin gene-related peptide (CGRP), stained small neurons in the medial part of the dorsal root ganglia from embryonic Day 5 and Day 10, respectively, whereas neurons in the lateral part of the ganglia were negative; this distribution persisted throughout development. Both sets of neurons apparently send fibers to the dorsal horn of the spinal cord: SP to laminae I and II, and CGRP to lamina I, suggesting that the SP- and CGRP-positive sensory neurons are nociceptive or thermoreceptive” (p. 337). Time point for onset of suffering cannot be inferred.		
19	Gottlieb et al.	1968	Ontogeny of vocalization in duck and chick embryos	Behavior	“Determine the earliest age at which duck and chick embryos are capable of vocalizing and the nature of the initial vocalizations”	“According to audiospectrographic analysis, both species are capable of uttering at least three different kinds of vocalizations **prior to hatching**, and these three calls are similar to the ones emitted most frequently after hatching “distress,” “contentment,” and “brooding-like calls” (p. 307). “[A]lmost all duck embryos could produce multiple notes by Day 24 and the same was true for chick embryos on **Day 19**” (p. 310). However, vocalizations were also evident in some embryos on **days 17 and 18** (p. 309).		
20	Corner et al.	1967	Developmental patterns in the central nervous system of birds. I. Electrical activity in the cerebral hemisphere, optic lobe and cerebellum	Review and cytology	“[S]ummarize at this time the known facts about normal central nervous development in birds (almost exclusively the chick)”	“Spontaneous slow waves appear in the hyperstriatum, optic tectum and cerebellum at the beginning of the third (last) week [**E14**] of incubation. Potentials can be elicited still earlier by a number of commonly used drugs” (p. 188). “The cerebral slow waves increase rapidly during *stage* 43 [**E16–17**] both in mean and peak amplitudes and in the frequency of their occurrence, and reach almost mature levels already at two to three days before hatching [**E18–19**]” (p. 188). “Electrical stimulation of the cerebral hemisphere, high temperature or treatment with strychnine, nembutal and many other drugs can elicit trains of large amplitude waves resembling sensory evoked-potentials” (p. 189).		
21	Schneider (48)	1961	Effects of morphine-like drugs in chicks.	Physiology/behavior	To describe the effects of morphine and similar drugs on nociception in chicks.	“[M]orphine and related drugs show only slight anti-nociceptive properties against acute mechanical stimuli in chicks, but cause postural and behavioral effects at low doses. Thus, in this species the side-effects of these drugs are more apparent than their anti-nociceptive action. The postural and other effects described appear to be characteristic of morphine and related drugs “(p. 608). Time point for onset of suffering cannot be inferred.		

Items are ordered chronologically. References to developmental days are in bold. Comments are added to the “key result” column where it was not possible to infer a timepoint for the commencement of capacity to suffer. Study reliability and relevance are indicated with red representing low; amber, medium; and green, high, respectively (see methodology for details).

## 3 Results and discussion

### 3.1 Essential characteristics of the shortlisted studies

Table 1 summarizes the results of the systematic review regarding the stage of life at which chicks begin to have the ability to suffer. Twenty-one relevant studies were located, comprising 6.5 reviews and 14.5 empirical studies. Most items (*n* = 18) were papers published in peer-reviewed journals, apart from one academic book chapter (23), one academic book (24), and one report from the science department of the German parliament (25). Over 50% of the shortlisted items were published after the year 2000. The 14.5 empirical studies comprised physiological (*n* = 3.5), behavioral (*n* = 2.5), and cytological/histological (*n* = 8.5) approaches, with the “0.5” number stemming from mixed-method studies. Of the 21 shortlisted items, only 10 had titles, aims, or results that were *explicitly* relevant to the key research question of the present report (i.e., those marked green in the Relevance column of Table 1). Nine of these 10 focused on pain/nociception rather than another aspect of the capacity to suffer, while one (26) focused on awareness, but without defining it. Five of these 10 most-relevant items explicitly defined pain using the IASP’s definition (see subsection 1, for details); another defined “nociceptor” briefly (27), while the remaining four gave no definition of pain or other terms related to suffering or welfare. This was also the case for the 11 less relevant items. See subsection 3.5 for a discussion of the reliability of the shortlisted items.

### 3.2 Key points of agreement

Among the shortlisted studies, there is consensus that the chicken species (*Gallus gallus domesticus*) is precocial. This means chicks reach an advanced stage of development before or at the time of hatching, as they are already relatively independent after hatching [e.g., (28), p. 243; (29), p. 153; (23), p. 44]. There is consensus that avian neuroanatomy is comparable to that of mammals. For instance, avians, like mammals, have a lateralized brain, meaning it is split into different areas with each having a more specialized role. Weiss et al. (30) and Douglas et al. (31) also mention how both C-fibers and A-delta fibers have been found in numerous parts of an avian’s body. These are collectively responsible for sensing generalized, chronic, and low-level pain, as well as acute sharp pain. Again, similar to mammals, avians possess high-threshold nociceptors capable of receiving different types of sensory input, which are part of the peripheral nervous system (31). Transduction occurs to transmit messages to the brain via the spinal-thalamic tract (32) (p. 0.9); (31) (p. 20). The neurotransmitter substance P (which plays a key role in pain signal transmission) that is found in mammals, is also found in avians, as are laminae I and II, which are cellular layers of the gray matter of the spinal cord that are responsible for receiving and modulating sensory input, respectively (31, 33).

One key neuroanatomical difference between avians and mammals is the lack of a neocortex in the avian forebrain (31). Nevertheless, the avian and mammalian forebrains (cerebrums) are still thought to function similarly, with the avian hyperpallium, nidopallium, and mesopallium being largely analogous to the mammalian neocortex (32) (p. 9). These two points—being precocial and having similar functional neuroanatomy to mammals—point to the capacity to suffer being developed in day-old chicks *at the very latest*. However, there is actually a broad consensus for the capacity to suffer commencing prior to hatching during the late stages of the 21-day incubation period—by “embryonic day” (E) 18 at the latest.

The evidence for the capacity to suffer having developed by E18 first centers around cytological (cell-based) and histological (tissue-based) evidence (see Table 2). Second, this has been further confirmed through the detection of the ability to feel pain or distress at different embryonic timepoints. Through different studies, these tests have provided holistic confirmation. The studies have included physiological measures, such as cardiovascular (30) and EEG [electroencephalogram; (32)]. They have also included behavioral measures, such as vocalizations (34) and variations in bodily movement in response to both noxious stimuli and pain relief (35). As demonstrated in Table 1, a likely starting point for the capacity to suffer was referenced in over half (*n* = 11) of the shortlisted items, either explicitly or by inference. Of these, nine supported capacity for suffering by E18 at the latest. Timepoints listed ranged through E13 (*n* = 1), E15 (*n* = 4), E17 (*n* = 1), and E18 (*n* = 3).

**Table 2:** Chronological overview of key neurodevelopmental milestones in chick embryos, by embryonic day (E).

ED	Nature of development	Key stage of development	Source	Relevance to suffering
<E3	Neuroanatomical	Neuritic processes begin	(41)	Extensions from neurons (e.g., axons) are required for transmission of pain signals to/from CNS
E3	Functional	Slight changes in heart rate first evident after change in gaseous concentration	(30)	Changes in heart rate is a common physiological change used as a possible indicator of stress or pain
	Behavioral	Spontaneous movements in chick embryo are evident	(35)	A possible precursor to conscious movement as an indicator of pain
	Neuroanatomical	First sensory neurons target dorsal root entry zone	(42)	This development is required for transmission of pain signals to the brain
E4	Neuroanatomical	Afferent nerve fibers develop	(30, 41, 43)	These carry pain signals from the PNS to the CNS
	Functional	Adrenergic/cholinergic receptors in heart are functional	(30)	Responsible for heart rate changes—a precursor to pain sensitization as pain affects the heart rate
E5	Neuroanatomical	Afferents detectable along entire descending/ascending tracts of the spinal column	(41)	Such neurogenesis is required for transmitting pain/distress signals to and from the brain
E6	Functional	Earliest EEG and sensory inputs into the spinal cord gray matter are detected	(30, 42)	Suggesting the beginnings of an active central nervous system
Consensus exists that pain is impossible before this point				
E7	Neuroanatomical	First synaptic connections are complete	(30, 44)	This is required for transmission of pain signals to and from the CNS
	Behavioral	First (reflex) behavioral responses to needle pinprick	(35)	A potential indicator of pain
E8	Neuroanatomical	Mass migration of proto nerve cells to the cerebrum begins	(32)	Intricate neural circuits start to be developed that could include pain perception
E9	Neuroanatomical	Projection of sensory inputs to laminae targets (an area of the spinal cord concentrated with neurons) detected	(42)	These laminae layers play a significant role in transmitting of sensory signals (including pain) to the CNS
E10	Neuroanatomical	Adrenergic tone is evident (sympathetic nervous system has an influence on blood pressure). Opioid receptors evident	(31)	Opioid receptors help to modulate pain and may indicate that pain is inhibited at this stage.
E11	Neuroanatomical	Completion of mass migration of neuroblasts	(28, 32)	Enables the development of intricate neural circuits including pain perception
E12	Neuroanatomical	Diencephalon has undergone full differentiation of nuclei	(32)	Brain areas become specialized for different roles, including pain perception
		Afferents likely able to activate spinal cord pain receptors at a time when laminae II become innervated	(27)	This would enable uninterrupted transmission of pain signals to the CNS
Ability to feel pain possible from this point				
E13	Neuroanatomical	Basic functional brain is developed	(30)	Meaningful development of the brain points to the potential for pain perception
		Dense projections into spinal cord are evident	(44)	Further meaningful development of neural pathways point to the potential for pain perception
	Functional	EEG is reliable in hyperpallium	(32, 45)	Indicates the potential for pain perception as the hyperpallium is the next target of sensory input to the thalamus
E14	Neuroanatomical	Spinal gray matter shows adult-like organization	(29)	This suggests potentially similar capabilities to adult chickens
E15	Functional	Nociceptive cardiovascular responses detected in at least some individuals	(30)	A potential indicator of pain
	Neuroanatomical	Peak in synaptic connections in forebrain	(23)	One of the key roles of the forebrain is that of pain perception
E16	Functional	Cerebral slow waves increase rapidly	(45)	Suggesting meaningful operational capacity of the cerebrum (part of the forebrain, which is key to pain perception)
E17	Behavioral	First detected coordinated movement	(35)	Indicative of consciousness and ability to respond to pain
Ability to feel pain highly likely from this point				
E18	Neuroanatomical	Complete differentiation of the central nervous system is finished	(36)	All neuroarchitecture is present with which to detect pain and feel distress
	Functional	Almost mature brain waves are reached	(45)	This suggests potentially similar capabilities to adult chickens
E19	Behavioral	Multiple notes akin to distress calls possible from now	(34)	A potential indicator not only of pain, but also other forms of distress
E20-21	Final few days prior to hatching on E21			

This table includes structural, functional, and sensory indicators relevant to the emergence of nociception and the potential capacity to suffer. Events are grouped by neuroanatomical development (in orange; e.g., neural tube formation), functional maturation (in blue; e.g., EEG activity), and behavioral reactivity (in green; e.g., movement). The citations are from the shortlisted studies of this systematic review. C/PNS refers to the central/peripheral nervous system. ED refers to embryonic day.

Some of the most important findings summarized in Table 1 include significant increases in mean arterial pressure (MAP) and heart rate in response to a noxious stimulus, relative to a control (30). These responses were detected at E16 and E17 respectively, and in both cases, the responses reduced after administration of pain relief. This demonstrates the importance of using multiple measures, as some may have different sensitivities to detecting nociception. There is also some evidence to suggest that pain sensitivity may differ in degree at different stages of development. For instance, Weiss et al. (30) found significant differences in MAP rises in response to a noxious stimulus at E16, but the response was stronger at E18. Additionally, Weiss et al. (30) highlighted that different readings in heart rate and MAP for different individuals demonstrate the individuality of pain perception (pp. 10–11).

Another key finding is that of Süß et al. (35). At E15, these authors found significantly more beak movements and, at E18, more leg/foot movements after a “pinch,” versus “little pinch” or “touch.” The authors also suggest that the significant differences in beak movement between the three levels of touch may indicate pain perception specifically, rather than broader nociception. Tellingly, they also found reduced head movements at E18 after application of pain relief. Kollmansperger et al. (32) found an even earlier sign of brain processing of nociceptive information, with EEG recordings demonstrating activity from E13.

While the capacity to suffer could likely develop at an earlier timepoint than E18, there is a broad consensus that it is near impossible for the capacity to suffer to emerge before E7 [e.g., (36), p. 112; (30), p. 2]. This is because, while the first afferent nerve fibers can be detected from E4, the earliest EEG readings are at E6.5 with the first synaptic connections being completed on E7. Exemplar stages in the developmental journey of a chick embryo are summarized in Table 2.

### 3.3 Key points of disagreement

Among the aforementioned 11 shortlisted items for which a starting point for the capacity to suffer is stated or can be inferred, there are two clear outliers. The authors of these outlier items point toward the capacity for suffering not beginning until the time of hatching or even later. They comprise a histological study by Necker (29) and a review by Mellor and Diesch (26). Necker (29) demonstrated that some pain modulating mechanisms are not fully developed until E20—just before hatching—suggesting that these systems are only required once the chick becomes independent. Mellor and Diesch (26) argued that, while chick embryos may have the neuronal capacity to experience awareness (and thus the capacity to suffer significantly) from E17, there are active neurosuppressors, such as adenosine, in play that maintain an unconscious state—including at the time of hatching and even immediately after. They state that the EEG readings support this too (p. 54). Another key concern Mellor and Diesch have is the reliance on nociception and extrapolation from mammals for inferring avian pain. Nociception comprises the physical/physiological aspects of pain, but not the emotional and subjective components (11)—i.e., it is a reaction to an aversive stimulus, rather than the perception of it. The authors deem this concern not only as a critique of individual papers/authors, but consider it a cultural/societal problem. Thus, there is concern about changing definitions of pain. This can be seen, for instance, in the paper by Süß et al. (35). These authors first introduce the definition by the IASP, but then proceed to state that, considering the problems of identifying pain without verbal report, pain could be defined as “a change in species-specific behavior as a possible consequence of a painful experience” (p. 2).

Some of these points are considered and countered by the other main cohort of authors. For instance, Kollmansperger et al. (32) contest that it is currently uncertain whether the embryonal electrical reading corresponds to a sleep-like state. Indeed, even Mellor and Diesch (26) themselves state that adenosine (neurosuppressor) levels are unknown in the chick (embryo). This could be an avenue of further investigation. Additionally, Koltzenburg and Lewin (27), among others, defend the use of nociception as one means of inferring pain in nonhuman animals. They contend that it is also necessary for inferring pain in nonverbal humans. Indeed, Douglas et al. (31) underscore that ascertaining pain in avians is particularly challenging because they are a prey species in which the flight response predominates. This means that overt signs of pain may be limited. Moreover, the same approaches (extrapolation from humans and use of nociception) are often used to assess pain in mammals (37), so it appears an inconsistency if this is permitted for mammals but not avians. Indeed, Weiss et al. (30) point out that MAP is a leading measure of nociception in mammals, as well as avians.

### 3.4 Implications of findings

National animal welfare legislation often excludes embryonic stages of life [e.g., England and Wales’ (38), s. 1.2]. This could be updated to reflect chick embryos’ potential to feel pain from embryonic day 13. This could affect hatchery practices such as the age at which relatively humane killing methods become important (e.g., urgent maceration versus discarding with other waste streams). It could also affect legislation in specialist areas. For instance, while the UK’s *Animals (Scientific Procedures) Act* (39) does currently cover chick embryos once they have reached the last third of incubation (s. 1.4.2), the time period covered could be extended to embryonic day 13 (roughly 2 days earlier). Emergent in-ovo sexing technologies are also increasingly being adopted to identify the sex of chick embryos prior to hatching (40). The intention of these technologies is to prevent the culling of newly hatched male chicks who are unwanted by the egg industry. Legislation should enforce their use by day 12 at the latest to specifically ensure that male chicks are destroyed prior to achieving sentience.

### 3.5 Limitations

Within any normal body of scientific evidence, various limitations are common. While no comprehensive reliability analysis of each included study was completed, the limitations of the items shortlisted within this systematic review mainly center around methodological choices and weaknesses in subsequent publications; hence, no item received a high (green) rating for reliability in Table 1. For instance, some studies only began examining embryo responses from E17 [e.g., (27)], meaning signs present at earlier embryonal stages could be missed. Some studies had very small sample sizes [e.g., (30)]. Some were exploratory studies only with no sophisticated power analyses [e.g., (30); p. 3]. Others sometimes failed to include sufficient detail in the methodology such as whether the first day of incubation counts as E0 or E1 (32) (p. 9). Such limitations should be corrected in future research. Finally, more research that has the development of the capacity for suffering in chickens as its core focus could further strengthen the scientific evidence base. This could also involve examining potential differences between different breeds as Kollmansperger et al. (32) suggested. Notions of suffering could also be broadened away from a heavy focus on pain, to include distress and discomfort, for instance.

## 4 Conclusion

Limitations of scientific evidence such as those identified in this review are normal within scientific studies. These do not negate the ability to draw overall conclusions with reasonable certainty, given the collected weight of scientific evidence. From this systematic review of relevant scientific studies, it is clear that there is a general scientific consensus that the capacity to suffer in chicks is likely to commence from late stages of incubation, specifically, by E18 and potentially as early as E13. This is indicated by cell- and tissue-based developments, in addition to EEG readings, maturation of neuroarchitecture, and physiological and behavioral responses to both noxious stimuli and pain relief. This is significant for the welfare of the 1.8 billion chicks hatched globally each month. National legislation should be updated to protect embryonated chicks.

## Data Availability

The original contributions presented in the study are included in the article/supplementary material, further inquiries can be directed to the corresponding author.
